# Anxiety and Depression Status of Patients With Coronary Atherosclerotic Heart Disease and Its Influencing Factors

**DOI:** 10.62641/aep.v53i6.2034

**Published:** 2025-12-17

**Authors:** Wenfeng He, Zhuang Shuai, Cheng Xue

**Affiliations:** ^1^Cardiovascular Disease Laboratory, Department of Cardiology, Affiliated Hospital of North Sichuan Medical College, 637000 Nanchong, Sichuan, China; ^2^School of Graduate Studies, Post Graduate Centre, Management and Science University (MSU), 40100 Shah Alam, Selangor, Malaysia

**Keywords:** coronary atherosclerotic heart disease, anxiety, depression, influencing factors

## Abstract

**Background:**

Coronary atherosclerotic heart disease (CAHD) is a major global health burden with high morbidity and mortality. Psychological comorbidities, particularly anxiety and depression, are highly prevalent in CAHD patients and significantly impact disease prognosis, quality of life, and treatment adherence. This study aimed to explore the occurrence and influencing factors of anxiety and depression in patients with CAHD.

**Methods:**

A retrospective study design was used to collect clinical data and questionnaire results from 152 patients with CAHD who attended our hospital from January 2022 to January 2025. The Hamilton Anxiety scale, Hamilton Depression scale, Acceptance of Illness Scale and Social Support Rating Scale were used to assess the results, and statistical analyses were performed using SPSS (version 26.0) software, which included independent sample *t*-tests, chi-square tests and univariate and multiple logistic regression analysis.

**Results:**

Amongst 152 patients with CAHD, the detection rate of anxiety symptoms was 42.76% (65 cases), and the detection rate of depressive symptoms was 46.05% (70 cases). Multiple logistic regression analysis showed that the number of coronary artery lesion branches (odds ratio (OR) = 3.15, 95% CI: 1.25–7.96, *p* = 0.015), the amount of long-term medication (OR = 3.26, 95% CI: 1.42–7.50, *p* = 0.005), and disease acceptance (OR = 0.81, 95% CI: 0.73–0.90, *p* < 0.001) and social support (OR = 0.88, 95% CI: 0.83–0.94, *p* < 0.001) were independent influencing factors of anxiety. Disease course (OR = 2.52, 95% CI: 1.18–5.41, *p* = 0.017), disease acceptance (OR = 0.92, 95% CI: 0.86–0.99, *p* = 0.047) and social support (OR = 0.95, 95% CI: 0.91–0.99, *p* = 0.047) were independent influencing factors of depression.

**Conclusion:**

Disease acceptance and social support are the main influencing factors. Therefore, routine screening for anxiety and depression, coupled with tailored interventions, is recommended for patients with CAHD.

## Introduction

Coronary atherosclerotic heart disease (CAHD) is a prevalent cardiovascular system disorder, characterised by the accumulation of atherosclerotic plaque in the arteries of the heart [1]. The global burden of CAHD is remarkable, with high morbidity and mortality rates, which pose a substantial threat to public health and life expectancy [2, 3]. With the biopsychosocial model, CAHD is gradually recognised to be not only a purely physical disease, but that psychological factors also play a crucial role in its occurrence, development and prognosis [4, 5]. Existing studies have shown that patients with CAHD are often accompanied by anxiety, depression and other adverse emotions, and these emotional disorders not only affect the mental health of patients but also pose a considerably negative effect on the therapeutic efficacy and prognosis of the disease [6]. These emotional disorders not only increase the distressing experience of patients but may also lead to decreased adherence to treatment and further exacerbation of cardiovascular disease [7]. Therefore, exploring the current status of anxiety and depression in patients with CAHD and analysing their influencing factors are of great value in improving patients’ conditions and quality of life.

Although anxiety and depression are prevalent in patients with CAHD, the mechanisms by which they occur are not fully understood. A combination of biological, psychological and social factors is now believed to be the main cause of anxiety and depression in patients with CAHD [8]. From a biological perspective, patients with CAHD are often associated with various cardiovascular risk factors such as hypertension, hyperlipidaemia and diabetes [9]. These factors not only affect the function of the cardiovascular system but may also increase the risk of psychological disorders in patients by affecting the metabolism of neurotransmitters in the brain and the regulation of the neuroendocrine system [10]. For example, prolonged myocardial ischaemia may lead to insufficient blood supply to the brain, affecting the synthesis and release of neurotransmitters, which, in turn, triggers anxiety and depression [11]. In addition, patients with CAHD may need to take multiple medications for a long period of time during the course of disease treatment, and the adverse effects of these medications may have a negative effect on the patients’ psychological state [12]. Patients with CAHD often face psychological stresses associated with the disease, such as fear of the disease, concern about the effectiveness of treatment and uncertainty about the future. These psychological stresses may lead to anxiety and depression in patients.

Currently, although studies have been conducted to explore the incidence of anxiety and depressed mood and their influencing factors in patients with CAHD, the findings have some differences. For instance, a cross-sectional study involving 414 Chinese patients found that the anxiety rate was 40.8% and the depression rate was 25.1%, whereas studies from other regions, such as Germany, have reported varying prevalence rates, underscoring the potential differences across populations and settings [13, 14]. In addition, significant differences may be present in the prevalence of anxiety and depressed mood and their influencing factors amongst patients with CAHD in different regions and populations. Therefore, the present study was conducted to analyse the current situation of anxiety and depression levels in patients with CAHD by retrospectively collecting patients’ data and questionnaires and to further explore their possible influencing factors. Against this background, the innovativeness of this study lies in two aspects. (1) Targeted outcome differentiation: Anxiety and depression were separately analysed as two distinct outcomes, and their respective independent influencing factors were identified. (2) Integration of objective and psychosocial factors: By incorporating disease-related objective indicators (e.g., coronary lesion severity and medication burden) and psychosocial factors (disease acceptance and social support) into the same analytical framework, the relative importance of these two types of factors was clarified. The results of this study could provide clinicians with a more comprehensive approach to assess the psychological state of patients with CAHD, thereby enabling early identification and intervention of anxiety and depression in patients with CAHD in clinical practice and improving their prognosis and quality of life.

## Materials and Methods

### Patient Population

A total of 152 patients with CAHD were included in this study using a retrospective study design. Their inclusion criteria were as follows: (a) meeting the diagnostic criteria and diagnosed with CAHD by laboratory tests [15]; (b) aged ≥18 years; (c) conscious, with normal communication and comprehension skills and able to cooperate in completing the questionnaires; and (d) with complete clinical information. The exclusion criteria were as follows: (a) comorbid serious neurological disorders, such as cerebrovascular disease and Parkinson’s disease, which may affect the assessment of mood; (b) suffering from serious psychiatric disorders such as schizophrenia and bipolar disorder; (c) presenting with other serious somatic disorders such as severe hepatic and renal insufficiency and malignant tumours; (d) received anti-anxiety and antidepressant medication in the last 3 months; and (e) experienced major life events in the last 3 months, such as widowhood and unemployment, which may have a strong and transient acute effect on mood. The study strictly adhered to all principles of the Declaration of Helsinki. The study protocol was approved by the Medical Ethics Committee of Affiliated Hospital of North Sichuan Medical College (Ethical Approval Number: 2025ER281-1). Informed consent was obtained from all patients.

### Data Collection of Patients

Demographic characteristics and disease-related information were collected by reviewing the patients’ electronic medical record system. The demographic characteristics included age, sex, marital status, education, smoking and alcohol consumption. The disease-related information included duration of coronary artery disease, type of coronary artery disease, Killip classification, number of diseased coronary arteries, percutaneous coronary intervention (PCI), long-term medication use and comorbidities (e.g., hypertension and diabetes mellitus). Additionally, following admission, healthcare professionals conducted routine psychological assessments on patients, and the information from these assessment scales was collected.

### Questionnaire Survey Scale

The Hamilton Anxiety (HAMA) scale was used to assess anxiety symptoms in patients with CAHD [16]. The scale was developed by Hamilton in 1959, and it contains 14 items covering somatic and psychogenic dimensions of anxiety. Each item is rated in accordance with the severity of symptoms, ranging from 0 to 4. The total score range of the HAMA scale is 0–56 points. A HAMA score of <7 indicates no anxiety symptoms, ≥7 indicates possible anxiety, ≥14 indicates definite anxiety symptoms and ≥21 indicates definite obvious anxiety. HAMA has good reliability and validity amongst the Chinese population with CAHD [17]. In the present study, a HAMA score of ≥7 was considered to have anxiety symptoms [18].

The Hamilton Depression (HAMD) scale was used to assess patients’ depressive symptom ratings [19]. The HAMD scale was developed by Hamilton in 1960. HAMD consists of 17 items assessing various dimensions of depression, including mood, guilt, insomnia and somatic symptoms. Each item of HAMD is given a score from 0 to 4 or from 0 to 2 in accordance with the severity of the symptom. The total score range of HAMD is 0–52 points. A HAMD score of <8 indicates no depression, ≥8 indicates mild depression, ≥17 indicates moderate depression and ≥24 indicates severe depression. The application of HAMD in the Chinese population has been verified [17]. In the present study, a HAMD score of ≥8 was considered to have depressive symptoms [18].

The Acceptance of Illness Scale (AIS) was used to assess patients’ cognitive and emotional acceptance of illness [20]. It consists of eight items assessing the patient’s cognitive and emotional acceptance of the disease on a scale of 1–5, with higher total scores indicating better acceptance.

The Social Support Rating Scale (SSRS) was developed by Xiao and Yang [21] in 1987 to assess the degree of social support received by individuals. The scale consists of three dimensions, namely, objective support, subjective support and use of social support, with a total of 10 items. SSRS uses a 1–4 or 1–3 rating system, with a total score range of 12–66. The higher the score on SSRS, the more social support an individual feels.

In this study, the Cronbach’α coefficients of the HAMA scale, the HAMD scale, AIS and SSRS were 0.81, 0.79, 0.85 and 0.91, respectively.

### Questionnaire Survey Method

All the questionnaire evaluations of the patients were saved in the electronic medical record system, and this information was retrospectively collected. The questionnaires were not administered at admission but after the patients’ condition stabilised and they had been hospitalised for 3–5 days to minimise the acute impact of the illness episode and the hospital environment on psychological assessment. This delay allowed for the resolution of the most acute physical distress and provided time for patients to adapt to the hospital setting, thereby capturing more of the underlying psychological status related to the chronicity of CAHD rather than the acute crisis. The researcher, who had undergone uniform training, explained to the patients the purpose, significance and method of filling in the survey and made sure that the patients understood the content of the questionnaire. Then, the patients filled in the questionnaire on their own. For patients with low literacy or those who could not fill in the questionnaire by themselves for other reasons, the researchers read out the questionnaire one by one and filled in the questionnaire on behalf of the patients in accordance with their answers. After the completion of the questionnaire, the researchers recovered the questionnaire on the spot and checked the completeness and logic of the questionnaire. If missing items or obvious errors were present, they communicated with the patients to make additions or corrections in a timely manner.

### Statistical Analysis

Data analysis was conducted using SPSS (version 26.0; IBM Corp., Armonk, NY, USA) and R (version 4.3.2; R Foundation for Statistical Computing, Vienna, Austria) software. The scores of each scale in this study all passed the normality test (Kolmogorov–Smirnov method). Continuous variables that conformed to normal distribution are expressed as mean ± standard deviation (SD). Independent sample *t*-test was used for the analysis of differences between groups. Categorical variables were expressed as frequency (n) and percentage (%), and chi-square tests were used to compare differences between groups. Univariate logistic regression analysis was used to preliminarily screen the influencing factors of anxiety and depression. Variables with *p* < 0.05 in the univariate logistic regression analysis were included in the multiple logistic regression model (backward stepwise) to select independent influencing factors (only statistically significant results were presented in the multiple logistic regression model). The discrimination of the logistic regression model was evaluated using the receiver operating characteristic (ROC) curve and the area under the curve (AUC). The variance inflation factor (VIF) was used to evaluate the collinearity amongst variables. VIF <5 is considered to have no collinearity problem. All the variables in the multiple analyses of this study passed the collinearity diagnosis. All statistical analyses were bilateral, and *p* < 0.05 was considered statistically significant.

## Results

### Anxiety and Depression in Patients With CAHD

A total of 152 patients with coronary artery disease were included in this study. The results showed that 65 patients had HAMA scores higher than the critical value, indicating that the detection rate of anxiety was 42.76% (Table 1). A total of 70 patients had HAMD scores higher than the critical value, that is, the detection rate of depression was 46.05%. The numbers of patients with mild anxiety and depression, as judged by scale scores, were 46 and 58, respectively, and the numbers of patients with moderate anxiety and depression were 19 and 12, respectively. No patients with severe anxiety nor depression were found.

**Table 1.** **Current situation of anxiety and depression in patients with CAHD**.

Group	Overall score	Negative	Mild	Moderate	Incidence rate
Anxiety^a^	7.44 ± 4.45	87 (4.29 ± 1.30)	46 (9.76 ± 2.04)	19 (16.26 ± 1.99)	42.76%
Depression^b^	8.78 ± 4.81	82 (5.04 ± 1.44)	58 (11.98 ± 2.38)	12 (18.83 ± 1.90)	46.05%

^a^HAMA <7: Negative; 7 ≤ HAMA < 14: Mild (possible anxiety); 14 ≤ HAMA < 21: Moderate (definite anxiety symptoms); HAMA ≥21: Severe (definite obvious anxiety). ^b^HAMD <8: Negative; 8 ≤ HAMD < 17: Mild; 17 ≤ HAMD < 23: Moderate; HAMD ≥23: Severe. Note: CAHD, coronary atherosclerotic heart disease; HAMA, Hamilton Anxiety Scale; HAMD, Hamilton Depression Scale.

### Distribution Differences of Anxiety Amongst Different Baseline Characteristics of Patients With CAHD

The results of univariate analysis showed that disease acceptance (*p* < 0.001), social support (*p* < 0.001), age (*p* = 0.018), marital status (*p* = 0.026), education (*p* = 0.032), diabetes mellitus (*p* = 0.017), duration of disease (*p* = 0.016), the number of branches of coronary artery lesions (*p* = 0.029) and the number of long-term medications (*p* = 0.005) were significantly associated with the occurrence of anxiety symptoms (Table 2).

**Table 2.** **Distribution differences of anxiety amongst different baseline characteristics of patients with CAHD**.

Variables		Total (n = 152)	Non-anxiety (n = 87)	Anxiety (n = 65)	Statistic	*p*
Disease acceptance, mean ± SD		26.61 ± 4.71	28.18 ± 4.43	24.51 ± 4.27	t = 5.14	<0.001
Social support, mean ± SD		40.83 ± 7.42	43.28 ± 7.31	37.55 ± 6.24	t = 5.08	<0.001
Age, n (%)					χ^2^ = 5.61	0.018
	<60 years	73 (48.03)	49 (56.32)	24 (36.92)		
	≥60 years	79 (51.97)	38 (43.68)	41 (63.08)		
Gender, n (%)					χ^2^ = 0.12	0.725
	Female	42 (27.63)	25 (28.74)	17 (26.15)		
	Male	110 (72.37)	62 (71.26)	48 (73.85)		
Marital status, n (%)					χ^2^ = 4.93	0.026
	Married	136 (89.47)	82 (94.25)	54 (83.08)		
	Other	16 (10.53)	5 (5.75)	11 (16.92)		
Degree of education, n (%)					χ^2^ = 4.59	0.032
	Senior high school and above	69 (45.39)	46 (52.87)	23 (35.38)		
	Junior high school and below	83 (54.61)	41 (47.13)	42 (64.62)		
Smoking, n (%)					χ^2^ = 0.65	0.421
	No	57 (37.50)	35 (40.23)	22 (33.85)		
	Yes	95 (62.50)	52 (59.77)	43 (66.15)		
Drinking, n (%)					χ^2^ = 1.02	0.314
	No	77 (50.66)	41 (47.13)	36 (55.38)		
	Yes	75 (49.34)	46 (52.87)	29 (44.62)		
Hypertension, n (%)					χ^2^ = 0.45	0.500
	No	105 (69.08)	62 (71.26)	43 (66.15)		
	Yes	47 (30.92)	25 (28.74)	22 (33.85)		
Diabetes, n (%)					χ^2^ = 5.69	0.017
	No	131 (86.18)	80 (91.95)	51 (78.46)		
	Yes	21 (13.82)	7 (8.05)	14 (21.54)		
CAHD classification, n (%)					χ^2^ = 0.90	0.342
	Stable coronary artery disease	109 (71.71)	65 (74.71)	44 (67.69)		
	Acute coronary syndrome	43 (28.29)	22 (25.29)	21 (32.31)		
Duration of CAHD, n (%)					χ^2^ = 5.79	0.016
	<5 years	109 (71.71)	69 (79.31)	40 (61.54)		
	≥5 years	43 (28.29)	18 (20.69)	25 (38.46)		
Killip classification, n (%)					χ^2^ = 2.25	0.133
	I, II	123 (80.92)	74 (85.06)	49 (75.38)		
	III, IV	29 (19.08)	13 (14.94)	16 (24.62)		
Number of lesion branches, n (%)					χ^2^ = 4.74	0.029
	Single	114 (75.00)	71 (81.61)	43 (66.15)		
	Double or more	38 (25.00)	16 (18.39)	22 (33.85)		
PCI surgery, n (%)					χ^2^ = 0.40	0.528
	No	45 (29.61)	24 (27.59)	21 (32.31)		
	Yes	107 (70.39)	63 (72.41)	44 (67.69)		
Number of long-term medications, n (%)					χ^2^ = 8.04	0.005
	<5	74 (48.68)	51 (58.62)	23 (35.38)		
	≥5	78 (51.32)	36 (41.38)	42 (64.62)		

Note: CAHD, coronary atherosclerotic heart disease; SD, standard deviation; PCI, percutaneous coronary intervention.

### Distribution Differences of Depression Amongst Different Baseline Characteristics of Patients With CAHD

The univariate analysis of depressive symptoms showed that disease acceptance (*p* = 0.004), social support (*p* = 0.008), duration of illness (*p* = 0.009) and the number of long-term medications (*p* = 0.048) were significantly associated with the occurrence of depressive symptoms (Table 3).

**Table 3.** **Distribution differences of depression amongst different baseline characteristics of patients with CAHD**.

Variables		Total (n = 152)	Depression (n = 82)	Non-depression (n = 70)	Statistic	*p*
Disease acceptance, mean ± SD		26.61 ± 4.71	27.61 ± 4.79	25.44 ± 4.37	t = 2.89	0.004
Social support, mean ± SD		40.83 ± 7.42	42.27 ± 7.93	39.14 ± 6.42	t = 2.68	0.008
Age, n (%)					χ^2^ = 2.26	0.133
	<60 years	73 (48.03)	44 (53.66)	29 (41.43)		
	≥60 years	79 (51.97)	38 (46.34)	41 (58.57)		
Gender, n (%)					χ^2^ = 0.73	0.394
	Female	42 (27.63)	25 (30.49)	17 (24.29)		
	Male	110 (72.37)	57 (69.51)	53 (75.71)		
Marital status, n (%)					χ^2^ = 1.95	0.163
	Married	136 (89.47)	76 (92.68)	60 (85.71)		
	Other	16 (10.53)	6 (7.32)	10 (14.29)		
Degree of education, n (%)					χ^2^ = 0.34	0.562
	Senior high school and above	69 (45.39)	39 (47.56)	30 (42.86)		
	Junior high school and below	83 (54.61)	43 (52.44)	40 (57.14)		
Smoking, n (%)					χ^2^ = 0.01	0.933
	No	57 (37.50)	31 (37.80)	26 (37.14)		
	Yes	95 (62.50)	51 (62.20)	44 (62.86)		
Drinking, n (%)					χ^2^ = 0.03	0.861
	No	77 (50.66)	41 (50.00)	36 (51.43)		
	Yes	75 (49.34)	41 (50.00)	34 (48.57)		
Hypertension, n (%)					χ^2^ = 0.02	0.900
	No	105 (69.08)	57 (69.51)	48 (68.57)		
	Yes	47 (30.92)	25 (30.49)	22 (31.43)		
Diabetes, n (%)					χ^2^ = 2.46	0.116
	No	131 (86.18)	74 (90.24)	57 (81.43)		
	Yes	21 (13.82)	8 (9.76)	13 (18.57)		
CAHD classification, n (%)					χ^2^ = 0.42	0.515
	Stable coronary artery disease	109 (71.71)	57 (69.51)	52 (74.29)		
	Acute coronary syndrome	43 (28.29)	25 (30.49)	18 (25.71)		
Duration of CAHD, n (%)					χ^2^ = 6.76	0.009
	<5 years	109 (71.71)	66 (80.49)	43 (61.43)		
	≥5 years	43 (28.29)	16 (19.51)	27 (38.57)		
Killip classification, n (%)					χ^2^ = 1.20	0.273
	I, II	123 (80.92)	69 (84.15)	54 (77.14)		
	III, IV	29 (19.08)	13 (15.85)	16 (22.86)		
Number of lesion branches, n (%)					χ^2^ = 1.73	0.188
	Single	114 (75.00)	65 (79.27)	49 (70.00)		
	Double or more	38 (25.00)	17 (20.73)	21 (30.00)		
PCI surgery, n (%)					χ^2^ = 0.01	0.922
	No	45 (29.61)	24 (29.27)	21 (30.00)		
	Yes	107 (70.39)	58 (70.73)	49 (70.00)		
Number of long-term medications, n (%)					χ^2^ = 3.92	0.048
	<5	74 (48.68)	46 (56.10)	28 (40.00)		
	≥5	78 (51.32)	36 (43.90)	42 (60.00)		

Note: CAHD, coronary atherosclerotic heart disease; SD, standard deviation; PCI, percutaneous coronary intervention.

### Univariate Logistic Regression Analysis of Anxiety and Depression in Patients With CAHD

The variable assignment process is shown in Table 4. The univariate logistic regression analysis showed that age (*p* = 0.019), marital status (*p* = 0.033), educational level (*p* = 0.033), diabetes (*p* = 0.021), disease duration (*p* = 0.017), the number of coronary artery lesion branches (*p* = 0.031), quantity of long-term medication (*p* = 0.005), disease acceptance (*p* < 0.001) and social support (*p* < 0.001) were risk factors for anxiety in patients with CAHD (Table 5). For depression, disease duration (*p* = 0.010), the number of long-term medications taken (*p* = 0.049), disease acceptance (*p* = 0.006) and social support (*p* = 0.011) were risk factors in patients with CAHD.

**Table 4.** **Variable assignment**.

Variables	Assignment
Disease acceptance	Original value
Social support	Original value
Age	<60: 0; ≥60: 1
Gender	Female: 0; Male: 1
Marital status	Married: 0; Other: 1
Degree of education	Senior high school and above: 0; Junior high school and below: 1
Smoking	No: 0; Yes: 1
Drinking	No: 0; Yes: 1
Hypertension	No: 0; Yes: 1
Diabetes	No: 0; Yes: 1
CAHD classification	Stable coronary artery disease: 0; Acute coronary syndrome: 1
Duration of CAHD	<5 years: 0; ≥5 years: 1
Killip classification	I, II: 0; III, IV: 1
Number of lesion branches	Single: 0; Double or more: 1
PCI surgery	No: 0; Yes: 1
Number of long-term medications	<5: 0; ≥5: 1

Note: CAHD, coronary atherosclerotic heart disease; SD, standard deviation; PCI, percutaneous coronary intervention.

**Table 5.** **Univariate logistic regression analysis of anxiety and depression in patients with CAHD**.

Variables		Anxiety		Depression	
		OR (95% CI)	*p*	OR (95% CI)	*p*
Age					
	<60 years	1.00 (Reference)		1.00 (Reference)	
	≥60 years	2.20 (1.14–4.25)	0.019	1.64 (0.86–3.12)	0.134
Gender					
	Female	1.00 (Reference)		1.00 (Reference)	
	Male	1.14 (0.55–2.34)	0.725	1.37 (0.67–2.81)	0.395
Marital status					
	Married	1.00 (Reference)		1.00 (Reference)	
	Other	3.34 (1.10–10.15)	0.033	2.11 (0.73–6.14)	0.170
Degree of education					
	Senior high school and above	1.00 (Reference)		1.00 (Reference)	
	Junior high school and below	2.05 (1.06–3.96)	0.033	1.21 (0.64–2.30)	0.562
Smoking					
	No	1.00 (Reference)		1.00 (Reference)	
	Yes	1.32 (0.67–2.57)	0.422	1.03 (0.53–1.99)	0.933
Drinking					
	No	1.00 (Reference)		1.00 (Reference)	
	Yes	0.72 (0.38–1.37)	0.314	0.94 (0.50–1.79)	0.861
Hypertension					
	No	1.00 (Reference)		1.00 (Reference)	
	Yes	1.27 (0.63–2.54)	0.500	1.04 (0.52–2.08)	0.900
Diabetes					
	No	1.00 (Reference)		1.00 (Reference)	
	Yes	3.14 (1.19–8.30)	0.021	2.11 (0.82–5.43)	0.122
CAHD classification					
	Stable coronary artery disease	1.00 (Reference)		1.00 (Reference)	
	Acute coronary syndrome	1.41 (0.69–2.87)	0.343	0.79 (0.39–1.61)	0.515
Duration of CAHD					
	<5 years	1.00 (Reference)		1.00 (Reference)	
	≥5 years	2.40 (1.17–4.92)	0.017	2.59 (1.25–5.36)	0.010
Killip classification					
	I, II	1.00 (Reference)		1.00 (Reference)	
	III, IV	1.86 (0.82–4.20)	0.137	1.57 (0.70–3.55)	0.276
Number of lesion branches					
	Single	1.00 (Reference)		1.00 (Reference)	
	Double or more	2.27 (1.08–4.79)	0.031	1.64 (0.78–3.43)	0.190
PCI surgery					
	No	1.00 (Reference)		1.00 (Reference)	
	Yes	0.80 (0.40–1.61)	0.528	0.97 (0.48–1.94)	0.922
Number of long-term medications					
	<5	1.00 (Reference)		1.00 (Reference)	
	≥5	2.59 (1.33–5.02)	0.005	1.92 (1.01–3.66)	0.049
Disease acceptance		0.83 (0.76–0.90)	<0.001	0.90 (0.84–0.97)	0.006
Social support		0.89 (0.84–0.94)	<0.001	0.94 (0.90–0.99)	0.011

Note: CAHD, coronary atherosclerotic heart disease; OR, odds ratio; CI, confidence interval; PCI, percutaneous coronary intervention.

### Multiple Logistic Regression Analysis of Anxiety Risk in Patients With CAHD

**Fig. 1.**
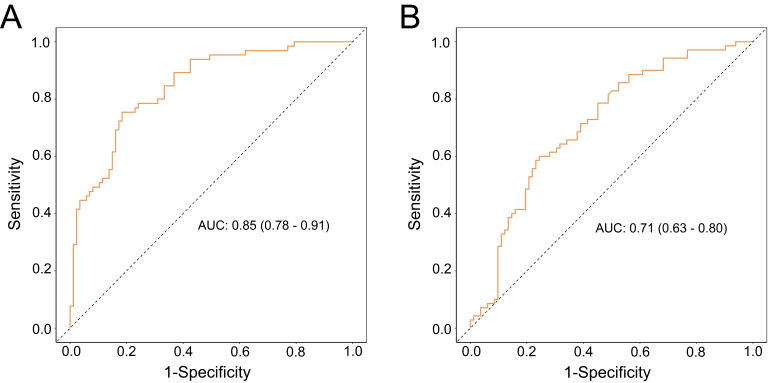
**ROC curve analysis**. (A) For anxiety. (B) For depression. Note: ROC, receiver operating characteristic; AUC, area under curve.

The multiple logistic regression analysis showed that the number of coronary artery lesion branches (OR = 3.15, 95% CI: 1.25–7.96, *p* = 0.015) and the amount of long-term medication (OR = 3.26, 95% CI: 1.42–7.50, *p* = 0.005) were independent risk factors for anxiety, whereas disease acceptance (OR = 0.81, 95% CI: 0.73–0.90, *p* < 0.001) and social support (OR = 0.88, 95% CI: 0.83–0.94, *p* < 0.001) were independent protective factors (Table 6). The ROC of this multiple regression model is shown in Fig. 1A. An AUC of 0.85 suggests that for any randomly selected pair of one patient with anxiety and one without, the model has an 85% chance of correctly identifying the patient with anxiety.

**Table 6.** **Multiple logistic regression analysis of anxiety in patients with CAHD**.

Variables		Β	SE	Z	*p * ^a^	OR (95% CI)
Number of lesion branches						
	Single					1.00 (Reference)
	Double or more	1.15	0.47	2.43	0.015	3.15 (1.25–7.96)
Number of long-term medications						
	<5					1.00 (Reference)
	≥5	1.18	0.42	2.78	0.005	3.26 (1.42–7.50)
Disease acceptance		–0.21	0.05	–3.94	<0.001	0.81 (0.73–0.90)
Social support		–0.13	0.03	–3.96	<0.001	0.88 (0.83–0.94)

^a^Confounding factors adjusted for in this multivariable model: age and gender. Note: CAHD, coronary atherosclerotic heart disease; SE, standard error; OR, odds ratio; CI, confidence interval.

### Multiple Logistic Regression Analysis of Depression Risk in Patients With CAHD

Disease duration (OR = 2.52, 95% CI: 1.18–5.41, *p* = 0.017) was an independent risk factor for depression, whereas disease acceptance (OR = 0.92, 95% CI: 0.86–0.99, *p* = 0.047) and social support (OR = 0.95, 95% CI: 0.91–0.99, *p* = 0.047) were independent protective factors for depression in patients with CAHD (Table 7). The ROC of this multiple regression model is shown in Fig. 1B. The AUC is 0.71, indicating that the accuracy rate of this model in distinguishing between patients with and without depression is 71%.

**Table 7.** **Multiple Logistic regression analysis of depression in patients with CAHD**.

Variables		Β	S.E	Z	*p * ^a^	OR (95% CI)
Duration of CAHD						
	<5 years					1.00 (Reference)
	≥5 years	0.93	0.39	2.38	0.017	2.52 (1.18–5.41)
Disease acceptance		–0.08	0.04	–1.99	0.047	0.92 (0.86–0.99)
Social support		–0.05	0.03	–1.99	0.047	0.95 (0.91–0.99)

^a^Confounding factors adjusted for in this multivariable model: age and gender. Note: CAHD, coronary atherosclerotic heart disease; SE, standard error; OR, odds ratio; CI, confidence interval.

## Discussion

CAHD is a cardiovascular disease that poses a serious threat to human health, and its high morbidity and mortality rates have become a significant burden on global public health [3]. In recent years, with the in-depth development of the biopsychosocial medical model, the important role of psychological factors in the occurrence, development and prognosis of CAHD has gradually received attention. This study retrospectively analysed the clinical and questionnaire data of 152 patients with CAHD and found that the detection rates of anxiety and depression were 42.76% and 46.05%, respectively. Previous studies have shown that the incidence of anxiety and depression amongst patients with CAHD can reach over 30%, which is consistent with the range of the results of the present study, highlighting the heavy burden of mental illness in this population [22].

In exploring the factors influencing anxiety in patients with CAHD, the study found that the number of branches of coronary artery disease, the number of long-term medications, disease acceptance and social support independently influence anxiety. The higher the number of coronary artery lesions, the higher the cardiovascular risk to the patients, which may trigger excessive worry about their health status and thus increase the risk of anxiety [23]. The long-term use of multiple medications can impose a considerable financial burden on patients, in addition to potentially causing physical discomfort due to adverse effects. These adverse effects can also have a detrimental effect on the patients’ psychological state, increasing their vulnerability to anxiety [24, 25]. The findings that low disease acceptance and inadequate social support are significant risk factors for anxiety and depression are consistent with the broader literature on chronic illness adjustment. For instance, a study by Dugunchi *et al*. [26] on patients with CAHD confirmed a strong correlation between poor illness perception and increased psychological distress. Similarly, a systematic review by Babygeetha and Devineni [27] concluded that robust social support is consistently associated with enhanced self-care and psychological well-being across various cardiac conditions, including heart failure. The results of the present study extend these established concepts by quantitatively demonstrating their independent protective effect against anxiety and depression in a well-defined CAHD cohort, even after controlling for clinical severity indicators. From a potential perspective, previous basic and clinical studies have suggested that psychological stress in patients with chronic cardiovascular diseases (including CAHD) may be associated with neuroendocrine and neurotransmitter-related changes. For example, activation of the sympathetic nervous system and hypothalamic-pituitary-adrenal (HPA) axis may disrupt neuroendocrine balance, and such changes have been linked to the occurrence of anxiety in cardiovascular disease populations [28]. However, a notable detail that the current study is a psychosocial questionnaire-based observational study, and indicators related to HPA axis activity or neurotransmitter metabolism were not measured. Therefore, a direct causal relationship between these physiological mechanisms and anxiety in patients with CAHD included in this study cannot be confirmed.

The results of the analysis of the factors influencing depressed mood showed that disease duration, disease acceptance and social support had independent influences on depressed mood in patients with CAHD. Patients with a long disease course suffer from the disease over a long period of time, their physical functions gradually decline and their quality of life could be seriously affected. These adverse effects could make patients prone to negative emotions, such as despair and helplessness, which, in turn, could lead to depression [29]. Patients with low disease acceptance are more likely to fall into fear and worry about the disease, and this prolonged state of psychological stress depletes their positive emotions and makes them more likely to fall into a depressed mood [26]. Inadequate social support can increase the risk of depressive moods by making it difficult for patients to relieve psychological stress due to lack of emotional support and practical help in the face of illness. Existing research has reported that long-term psychological stress in patients with cardiovascular diseases may be related to changes in brain structure and function (e.g., reduced hippocampal volume and abnormal prefrontal cortex function) and inflammatory response activation, and these changes have been proposed to be associated with the development of depressive mood by affecting emotional regulation and neurotransmitter metabolism [30, 31, 32]. However, the current study used questionnaires to assess psychological status and clinical characteristics only, without collecting objective biological indicators. As a result, direct evidence for the involvement of hippocampal structural changes, neurotransmitter metabolism disorders or inflammatory activation in the occurrence of depression in patients with CAHD cannot be provided in this study. The abovementioned potential mechanisms are references to existing research conclusions, and their applicability to the study population needs to be validated in future studies that integrate psychosocial assessments with biological measurements.

The findings provide a strong rationale for integrating psychosocial interventions into standard cardiac care. The protective role of disease acceptance suggests that interventions grounded in cognitive-behavioural therapy (CBT) and acceptance and commitment therapy (ACT) could be beneficial. This view is supported by a Cochrane review by Ski *et al*. [33], who found that psychological interventions, particularly CBT, are effective in reducing depression and anxiety in patients with coronary heart disease. Specifically, structured sessions to help patients reframe negative thoughts about their illness, as suggested by the data in the present study, could be a core component. Similarly, facilitating patient support groups to bolster the protective effect of social support aligns with recommendations found in literature [27]. Family-centred interventions that educate and involve patients’ families could further strengthen the support system. Integrating these evidence-based psychological and social strategies into standard cardiac rehabilitation programs could provide a holistic approach to patient care, ultimately improving mental health and cardiovascular outcomes [33].

However, this study has some limitations. Firstly, this study adopted a retrospective research design, which may have selection and information biases, thus affecting the accuracy and reliability of the findings. Secondly, the sample size was relatively small and limited to patient groups in a single region, which may not comprehensively reflect the occurrence of anxiety and depression and their influencing factors in patients with CAHD in different regions and populations, thereby limiting the extrapolation of the study results. Thirdly, the selection of variables for the multiple model was based on a univariate screening threshold (*p* < 0.05), which is a common approach for exploratory studies but carries the risk of missing potential confounders that are clinically relevant but not statistically significant in univariate analysis. Future studies could consider a different approach such as including established clinical risk factors a priori regardless of their univariate *p*-value. In addition, this study only assessed patients’ anxiety and depression by means of questionnaires. It lacked more objective biological indexes, such as neurotransmitter levels and gene polymorphisms, which made it difficult to explore the mechanisms of anxiety and depression in depth from the level of biological mechanisms. Future studies can adopt a prospective study design to expand the sample size and include patients with CAHD from different regions and populations to improve the accuracy and representativeness of the study results. Moreover, biological tests, such as neurotransmitter levels in patients’ blood and gene polymorphisms, can be used to explore the mechanisms of anxiety and depression from a multidimensional perspective and provide a more scientific basis for clinical intervention.

## Conclusion

This study confirms a high prevalence of anxiety and depression amongst patients with CAHD, underscoring the necessity of routine psychological screening in this population. Distinct profiles of modifiable and non-modifiable influencing factors for these conditions were identified. Specifically, the severity of coronary lesions and high medication burden were independent risk factors for anxiety, and lengthened disease duration increased the risk for depression. Crucially and of paramount clinical relevance, strengthened disease acceptance and social support served as significant protective factors against anxiety and depression. These findings highlight a critical shift in patient management: moving beyond purely biological treatment to integrate psychosocial care. Clinicians should prioritise assessing and enhancing patients’ understanding and acceptance of their illness whilst fostering robust social support systems. Interventions targeting these modifiable factors, such as incorporating principles from CBT and facilitating support groups, hold great promise for mitigating psychological distress and potentially improving overall cardiovascular outcomes in patients with CAHD.

## Availability of Data and Materials

All experimental data included in this study can be obtained by contacting the first author if needed.
